# Faunistic Contributions to the Superfamilies Oestroidea and Muscoidea (Insecta: Diptera) of Greece and Cyprus: New Records from Five Calyptrate Families

**DOI:** 10.3390/insects17040433

**Published:** 2026-04-17

**Authors:** Gabriella Dimitra Rakopoulou, Savvas Zafeiriou, Nikoleta-Nefeli Kofou, Theodora Petanidou, Georgios Agapakis

**Affiliations:** 1 Entomology and Nematology Department, University of Florida, Gainesville, FL 32611, USA; 2 Laboratory of Biogeography and Ecology, Department of Geography, University of the Aegean, University Hill, 81100 Mytilene, Greece; savzafi@yahoo.com (S.Z.); nkofou@geo.aegean.gr (N.-N.K.); tpet@aegean.gr (T.P.); 3 Laboratory of Spatial Analysis Geographic Information Systems and Remote Sensing (SAGISRS), Department of Geography, University of the Aegean, University Hill, 81100 Mytilene, Greece; 4 Department of Biotechnology, Agricultural University of Athens, 11855 Athens, Greece; gagapakes@gmail.com

**Keywords:** Calyptratae, Muscoidea, Oestroidea, faunistics, biodiversity, distribution, checklist, new records, Greece, Cyprus

## Abstract

Knowledge of the Oestroidea and Muscoidea fauna of Greece and Cyprus remains fragmentary, with substantial parts of the two countries having never been systematically surveyed. The present study verifies the presence of *Scathophaga stercoraria* (Linnaeus, 1758) in Cyprus and records 16 new species from Greece, belonging to five calyptrate families: [*Anthomyia illocata* Walker, 1856 (Muscoidea: Anthomyiidae); *Scathophaga lutaria* (Fabricius, 1794) (Muscoidea: Anthomyiidae); *Fannia pallitibia* (Rondani, 1866); *Fannia pusio* (Wiedemann, 1830) (Muscoidea: Fanniidae); and *Coenosia* sp. nov. 1, *Coenosia* sp. nov. 2, *Lispe flavicincta* Loew, 1847, *Lispe nuba* Wiedemann, 1830, *Lispe orientalis* Wiedemann, 1824, *Lispe* cf. *sericipalpis* (Stein, 1904), *Potamia littoralis* Robineau–Desvoidy, 1830 (Muscoidea: Muscidae); *Apodacra radchenkoi* Verves and Khrokalo, 2015,* Craticulina tabaniformis* (Fabricius, 1805), *Miltogramma rutilans* Meigen, 1824, *Nyctia lugubris* (Macquart, 1843) (Oestroidea: Sarcophagidae), and *Linnaemya lithosiophaga* (Rondani, 1859) (Oestroidea: Tachinidae)]. These records are based on the examination of 152 dry-pinned specimens from 58 localities, collected between 1978 and 2026 across Greece and Cyprus using a combination of passive (animal-baited traps, UV-bright pan traps) and active (hand collecting, net sweeping) sampling methods, together with insect material from the entomological collections of the National Museum of Natural History Goulandris and the Melissotheque of the Aegean. In addition, the first checklists of the family Fanniidae and the subfamily Scathophaginae for Greece and Cyprus are presented. Collectively, the findings presented expand the documented diversity of Greek and Cypriot Calyptratae and refine the current understanding of their biogeographic patterns, providing an updated framework for taxonomic, ecological, forensic, and other applied entomological research within the two countries.

## Simple Summary

Calyptrate flies of the superfamilies Oestroidea and Muscoidea (Insecta: Diptera) are among the most important dipteran groups from a medical, forensic, veterinary, and ecological perspective. Despite their significance, these groups in Greece and Cyprus remain inadequately documented and geographically fragmented. The present study summarizes results from recent field surveys and examination of entomological material housed in the insect collections of the National Museum of Natural History Goulandris and the Melissotheque of the Aegean from 1978 to 2026. Specimens were collected from 58 distinct localities across Greece and Cyprus using both passive (animal-baited traps, UV-bright pan traps) and active (net sweeping, hand collecting) sampling methods. Sixteen species belonging to five families were identified as new records for Greece. This study provides the first checklist of the family Fanniidae and the subfamily Scathophaginae for Greece and Cyprus. The records add to the distribution, refine the known composition of Greek and Cypriot calyptrate Diptera fauna, and provide an updated baseline for future research in these countries.

## 1. Introduction

The superfamilies Oestroidea and Muscoidea (Insecta: Diptera) represent a diverse and ecologically important group of calyptrate Diptera [1]. Species within these taxonomic groups occupy a wide range of ecological niches, ranging from saprophagous [2] and coprophagous taxa associated with decomposing organic matter and dung [3] to pathogen vectors [4], haematophagous species, and parasitoid taxa [5]. These species are of medical, forensic, veterinary, and agricultural importance [3,6,7,8,9,10,11,12,13]. The larvae of these species are involved in nutrient recycling and decomposition processes [14]. On the other hand, adults are known to participate in pollination [15] and act as prey or predators [16,17] within complex trophic webs. As a consequence of these varied ecological functions and their sensitivity to environmental conditions, certain oestroid and muscoid flies may be used as indicators in biodiversity assessments and in applied contexts such as forensic entomology [18,19], livestock health [20], and pest management [21].

Within Muscoidea, families such as Muscidae (House Flies), Fanniidae (Lesser House Flies), and Anthomyiidae (Root-maggot Flies) include many widespread, synanthropic, and rural-associated flies worldwide [4]. Numerous species belonging to the families Muscidae and Fanniidae are closely linked to animal husbandry, public health, and human-modified environments [22]. Flies within Anthomyiidae are particularly associated with grassland, wetland, and agricultural habitats, often found in soil, dung, or plant tissue [23,24]. Oestroidea, by contrast, is dominated by taxa with saprophagous, necrophagous, or parasitoid life histories, notably within the families Tachinidae (Bristle Flies) and Sarcophagidae (Flesh Flies) [25,26,27]. Tachinid flies constitute one of the largest families of parasitoid insects and are of importance as natural enemies of Lepidoptera [28] and other phytophagous pests [29]. Sarcophagidae comprise an ecologically diverse group, including numerous kleptoparasitic Miltogramminae [30], several parasitic or parasitoid representatives from the subfamilies Sarcophaginae and Paramacronychiinae [31,32], and some species associated with carrion and vertebrate remains [9] that have documented forensic relevance [33]. Despite their ecological significance, the fauna of these families remains incompletely documented and not well understood in many parts of the Palaearctic, including the eastern Mediterranean [34].

Existing scattered records in Greece and Cyprus have revealed a diverse assemblage of oestroid and muscoid flies [3,7,8,9,35,36,37,38,39,40,41,42]. The variable documentation of these taxonomic groups potentially obscures patterns of species richness and endemism in the Greek and Cypriot regions, and in turn, this may limit the resolution of biogeographic, ecological, and applied studies that rely on such baseline data. This heterogeneity in the current state of knowledge is also reflected in the families treated in the present study, as Anthomyiidae (including Scathophaginae), with 96 species recorded from Greece, and Fanniidae, with 17 recorded species, remain among the least documented families, both in terms of known diversity and the lack of extensive, targeted studies in the country [3,7,8,43,44,45,46,47,48,49,50,51]. By contrast, Tachinidae is the most species-rich family, with 347 species, but it still lacks a proper country-wide checklist [38,43,52,53]. Similarly, Sarcophagidae has 109 recorded species in Greece, with only the genus *Sarcophaga* being studied [9], while records of the remaining genera are dispersed across the literature [43,54,55,56,57]. Notably, Muscidae, with 160 species recorded to date, is the only family among those treated here for which a relatively recent country-wide synthesis is available [39], further supplemented by additional records published in individual studies [3,42,58,59,60]. Finally, numerous species belonging to the aforementioned families and recorded from neighbouring countries [61,62,63,64,65,66,67,68] remain unrecorded at the national level.

Against this backdrop, the present contribution aims to document previously unreported Oestroidea and Muscoidea from Greece and Cyprus and includes new Greek records for species in five families: Anthomyiidae, Fanniidae, Muscidae, Sarcophagidae, and Tachinidae, thereby expanding and validating the known composition of the Greek and Cypriot calyptrate fauna. The data presented add to the current knowledge of the diversity and distribution of these groups and provide an updated reference framework for subsequent taxonomic, faunistic, forensic, and ecological research in the two countries.

## 2. Materials and Methods

### 2.1. Methodology

The present study concerns the examination of 73 museum and 79 field-collected specimens obtained between 1978 and 2026 from 58 geographically dispersed localities across Greece and Cyprus. Fly material was obtained through a combined sampling methodology, integrating both passive and active collection techniques. Passive sampling consisted of the deployment of baited bottles and pitfall traps provisioned with a proteinaceous animal bait and operated in accordance with the methodology outlined in Rakopoulou and Dadour [8]. In addition, insects deposited in the Melissotheque of the Aegean (M.A.) were collected from Cyprus, several Greek islands, and mountain sites using the pan trap and hand-netting protocol [69,70,71,72]. At least three visits to each site were conducted during the main flowering season, which incorporated ten pan trap triplets. Each triplet consisted of three UV-bright pan traps of yellow, blue, and white colours. Each pan trap was filled with 350 mL of water in which one drop of aroma-free detergent was added and left onsite for 48 h prior to collection. Active sampling was undertaken by means of sweep netting and manual collection, enabling the targeted capture of adult individuals encountered in the field. In addition to newly collected material, specimens deposited in the entomological collection of the National Museum of Natural History Goulandris (N.M.N.H.G.) were examined.

**Figure 1:**
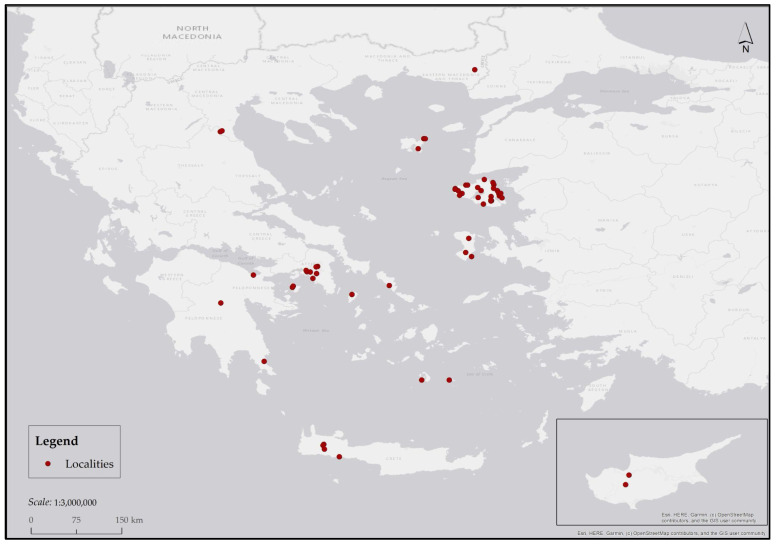
Map depicting all studied localities in Greece and Cyprus.

In the laboratory, all specimens were pinned, enumerated, and sexed. Taxonomic determinations to species level were carried out under a stereo microscope (ZEISS Stemi 508 doc; Carl Zeiss Microscopy GmbH, Göttingen, Germany; S/N 3951008128; Belarus), with each identification corroborated using specialized taxonomic keys and publications [45,47,48,54,73,74,75,76,77,78,79]. Data on European distributional ranges for the recorded taxa were compiled from multiple bibliographic sources [43,46,48,49,52,57,76,78,80,81]. The nomenclature and taxonomic classification adopted herein follow Buenaventura et al. [32] for Oestroidea, Gregor et al. [76] for Muscidae, Domínguez and Roig-Juñent [82] for Fanniidae, and the revised phylogenetic framework of Bailey et al. [83] for root-maggot flies (Anthomyiidae) and dung flies (formerly Scathophagidae). Geographic coordinates and associated locality information for all sampling sites are provided in Table 1. Spatial distribution of these localities is depicted in Figure 1. All maps were generated using ArcMap 10.7.1. The material examined in the present study is currently deposited in the private collections of the contributing authors (G.D.R., S.Z., G.A.) and curated within the institutional repositories of the M.A., Mytilene, Greece, and the N.M.N.H.G., Athens, Greece.

### 2.2. Format

Results are presented as a faunistic synthesis incorporating new records, supplemented by systematic checklists of the family Fanniidae and the subfamily Scathophaginae for Greece and Cyprus. Taxa are arranged by superfamily and family, and within each family, species are listed in alphabetical order. Material available for examination is presented under “Material examined”. Species records are formatted by providing the country and the administrative unit and/or island in uppercase and underlined, the collecting locality, the number and sex of specimens, and the date of collection; separate collecting events are delimited by semicolons. Occurrence in Greece is subsequently summarized for each species under “Distribution in Greece” and supported by bibliographic sources. A “Distribution” statement follows, summarizing the general range and citing the pertinent references. A “Comments” section is provided for taxa, including diagnostic or taxonomic considerations, uncertain status of nominal taxa, faunistic significance of particular occurrences, or biologically/ecologically relevant notes directly linked to the material examined. A second component presents faunistic records from additional muscoid and oestroid families. These records are arranged by family and presented in a comparable format to the systematic checklist. For taxa that could not be assigned securely to the species level, the rationale for this treatment is documented in “Comments”, including diagnostic limitations, uncertainty arising from the available material, or indications of potentially undescribed taxa; where relevant, the “Comments” section also provides pertinent biological/ecological notes and broader significance. Finally, in both parts, newly added species for Greece are marked with a black triangle (^▲^), while new records within the country are indicated with an asterisk (*).

## 3. Results

**Figure 2:**
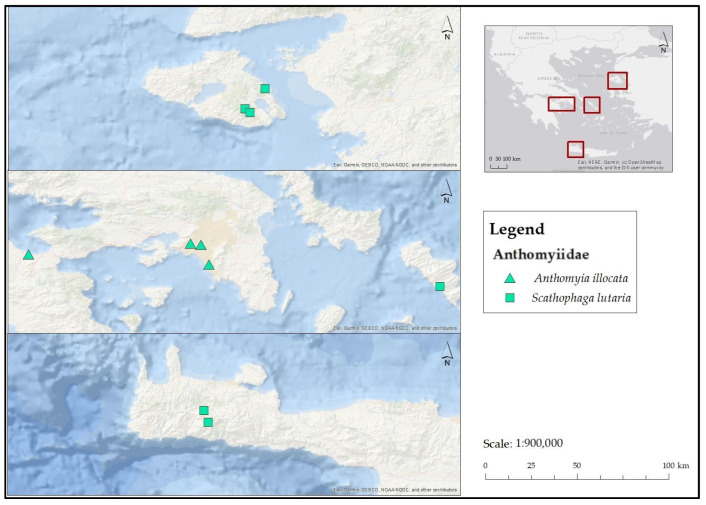
Map depicting collection localities in Greece where *A. illocata* and *S. lutaria* were recorded during the present study.

**Figure 3:**
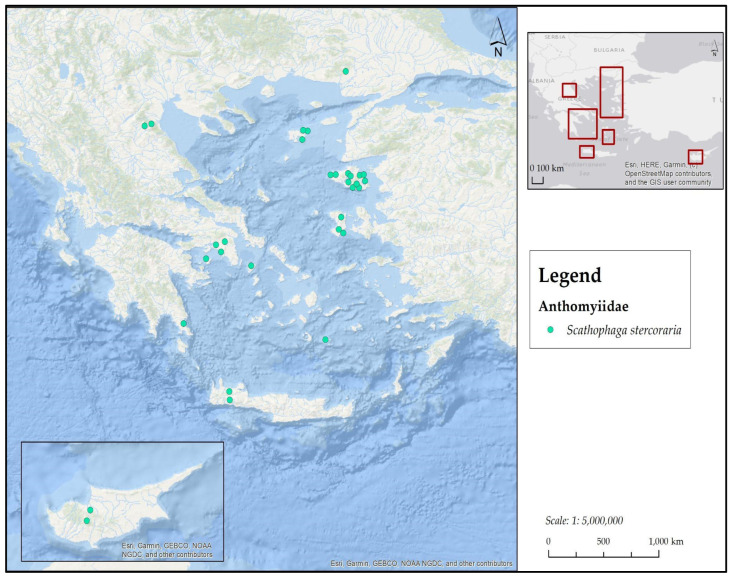
Map depicting collection localities in Greece and Cyprus where *S. stercoraria* was recorded during the present study.

**Superfamily Muscoidea**
**Family Anthomyiidae (** **Figure 2 and Figure 3** **)**
***Anthomyia illocata* Walker, 1856*^▲^* (Figure 7H)**
Material examined: GREECE*: ATTIKI*: Agia Varvara, 2♀, 15 May 2017 (G.A.); 1♂, 5 June 2022 (G.A.); 1♂, 4 April 2023 (G.A.); Agricultural University of Athens, 1♀, 30 May 2021 (G.D.R.); 1♂ & 1♀, 14 June 2021 (G.D.R.); 1♂ & 3♀, 28 June 2021 (G.D.R.); 1♂, 8 July 2021 (G.D.R.); 1♀, 16 July 2021 (G.D.R.); Ellinikon International Airport, 1♂, 9 April 2023 (S.Z.); KORINTHIA*: Lechaio: 1♂, 18 July 2023 (G.A.); 1♀, 26 July 2023 (G.A.); LESVOS*: Mytilene, 1♂, 12 October 2023 (S.Z.).
**Distribution:** A synanthropic species, widespread in the East Palaearctic, Oriental, Australasian, and Oceanian regions [79]. New for Greece and Europe.
**Comments:** Larvae are primarily coprophagous, developing in fecal substrates [84]. Adults are commonly associated with a range of dung types and carrion and may serve as potential mechanical vectors, facilitating the transfer of pathogenic microorganisms across habitats and thereby contributing to the dissemination of disease agents in different environments [85,86,87]. Based on uploaded photographs of specimens bearing the characteristic chromatic pattern of *A. illocata* from various citizen science sites (e.g., iNaturalist), this species seems to have been introduced to various places in Europe. As yet, no research has been published concerning these records. The specimens examined here constitute the first official recording of the species in Europe.
***Norellia spinipes* (Meigen, 1826)**
**Distribution in Greece:** Known from the provinces of Attiki, Cyclades (Iraklia Island), Ionian Islands (Corfu), and Peloponnese (Mount Taygetus) [7,46].
**Distribution:** Widespread, but with scattered records in Europe. Also present in North Africa (Algeria) [46].
**Comments:** Reported to develop in the leaves of daffodil (*Narcissus pseudonarcissus* L.) in dune forests [88].
***Scathophaga ?furcata* (Say, 1832)**
**Distribution:** Widespread in the Holarctic region [45].
**Comments:** A species written as “*Scatophaga synalida* Mg.” was reported from the island of Cyprus in 1977 [35]. No valid or synonymised name exists for *Scathophaga* with “*synalida*” as its specific part, belonging to Meigen (=Mg.) [45,46]. However, there is the name *Scathophaga squalida* (Meigen, 1826), which is considered a junior synonym of *S. furcata* [45,46]. Therefore, it is possible that Georghiou’s [35] “*Scatophaga synalida*” is just a misspelling of *S. squalida* (=*S. furcata*). There is no new or museum material of *S. furcata* available from Cyprus. As a consequence, with no comparable specimens of “*synalida*”, the true identity of these specimens remains unknown.
***Scathophaga lutaria* (Fabricius, 1794)*^▲^* (Figure 7E)**
Material examined: GREECE*: ANDROS*: Zaganiaris, 1♀, 6 April 2015 (M.A.); CHANIA*: Omalos I, 1♀, 28 March 2023 (M.A.); Omalos II, 1♂, 28 March 2023 (M.A.); 8♂ & 13♀, 31 August 2023 (M.A.); 1♀, 26 September 2023 (M.A.); LESVOS*: Pirgi Thermis, 1♀, 21 April 2020 (S.Z.); Radar Agiasou, 1♀, 28 September 2024 (S.Z.); Sanatorio Agiasou, 1♀, 28 April 2022 (S.Z.); 1♂, 8 October 2022 (S.Z.); 1♂ & 1♀, 8 July 2023 (S.Z.); 1♀, 13 July 2024 (S.Z.); 2♂, 2 August 2025 (S.Z.).
**Distribution:** Widespread in the Holarctic region [80]. New for Greece.
**Comments:** Larvae are carnivorous in dung and in rotten seaweed [89]. The species was first reported from the island of Cyprus in 1977 [35] but has been omitted from all major databases and catalogues concerning the family [43,46]. Since no new or museum material was available, we were unable to verify its presence on the island.
***Scathophaga ochrocephala* (Brullé, 1832)**
**Distribution in Greece:** Known from mainland Greece without specific localities [46].
**Distribution:** Up to now, known only from Greece [46].
**Comments:** The validity of this species remains unclear, as the depository of the type and only specimens remains unknown, with nothing recorded since the original description [46].
***Scathophaga stercoraria* (Linnaeus, 1758) (Figure 7F)**
**Material examined: CYPRUS:** LEMESOS*: Peak of Troodos Mountain, 3♀, 16 May 2012 (M.A.); NICOSIA*: Linou, 1♂, 7 April 2012 (M.A.); **GREECE:** AEGINA*: Kavouropetra, 1♂, 26 September 2012 (M.A.); ANAFI*: Helicodrome, 1♀, 12 May 2013 (M.A.); ATTIKI: Alsos Syggrou, 2♂ & 1♀, 27 March 2022 (S.Z.); Diomedes Botanical Garden, 1♂, 26 March 1986 (N.M.N.H.G.); Ellinikon International Airport, 1♀, 5 February 2022 (S.Z.); CHANIA*: Kefali, 1♀, 28 March 2023 (M.A.); Omalos II, 1♀, 28 March 2023 (M.A.); CHIOS*: Gridia, 2♂, 22 May 2022 (M.A.); Pityos, 1♂, 31 March 2012 (M.A.); Vessa, 1♂, 21 May 2022 (M.A.); EVROS*: Dadia, 1♂, 29 April 2013 (M.A.); KEA*: Mylopotamos, 1♂, 23 April 2013 (M.A.); LAKONIA*: Agios Stefanos, 2♂, 29 March 1978 (N.M.N.H.G.); 1♂, 1 May 1978 (N.M.N.H.G.); LESVOS*: Chestnut Forest I, 1♀, 27 April 2021 (M.A.); 1♂, 26 May 2021 (M.A.); Chestnut Forest II, 1♂, 23 June 2004 (M.A.); Chestnut Forest III, 1♂ & 2♀, 8 May 2009 (M.A.); Kalloni Saltpans, 1♂, 7 April 2005 (M.A.); Kamaria Pamfilon, 1♂, 21 April 2023 (S.Z.); 1♂, 23 March 2024 (S.Z.); Lisvori, 1♂, 19 April 2007 (M.A.); Moria, 1♂, 16 April 2005 (M.A.); Panagia Mirsiniotissa, 1♀, 15 February 2026 (S.Z.); Petrified Forest Park “Bali Alonia′′, 1♀, 19 June 2011 (M.A.); Pirgi Thermis, 2♀, 3 December 2023 (S.Z.); 1♂, 28 March 2025 (S.Z.); 1♂, 6 April 2025 (S.Z.); Sanatorio Agiasou, 1♂, 12 July 2020 (S.Z.); Sigri, 1♂, 30 March 2012 (M.A.); LIMNOS*: Moudros, 2♂, 6 April 2012 (M.A.); Panagia, 1♀, 8 April 2012 (M.A.); Plaka-Panagia, 3♂ & 7♀, 5 April 2012 (M.A.); PIERIA*: Oropedio Olympou, 1♂, 9 August 2014 (M.A.); Robola Olympou, 1♂, 15 September 2013 (M.A.).
**Distribution in Greece:** Definitely known from the provinces of Attiki (only mainland part) and Cyclades (Iraklia Island) [3,7,8,90]. Previously reported from the country, but without specific localities [45]. New for Thrace, North Aegean Islands, East Aegean Islands, Anafi Island (Cyclades), Aegina Island (Attiki), Peloponnese, and Crete.
**Distribution:** Widespread in the Holarctic region, with scattered records also from the Afrotropical and Oriental regions [80].
**Comments:** Primarily associated with the dung of large mammals, particularly livestock [91]. Extensively used as a model organism in ecological, behavioural, and evolutionary research [92]; also recorded in carrion decomposition [93] and baited trap experiments [3,8]. The species was reported as present in Cyprus in 1977 [35], but was later omitted from all major databases and catalogues concerning the family [43,46]. The examination of museum material by the authors verifies its presence on the island.
***Scathophaga taeniata* Rondani, 1866**
**Distribution:** Scatteredly recorded from a few places in the Palaearctic (Western Europe, Russia, North China) and Nearctic regions (Canada) [45].
**Comments:** The species is commonly treated as a synonym of *S. suilla* (Fabricius, 1794), so the true extent of its distribution is unknown [45]. Reported, under its synonym *S. ordinata* (Becker, 1894), from the island of Cyprus since 1977 [35], but omitted from all major databases and catalogues concerning the family [43,46]. Since no new or museum material is available for examination, verification of its presence on the island was impossible.

**Figure 4:**
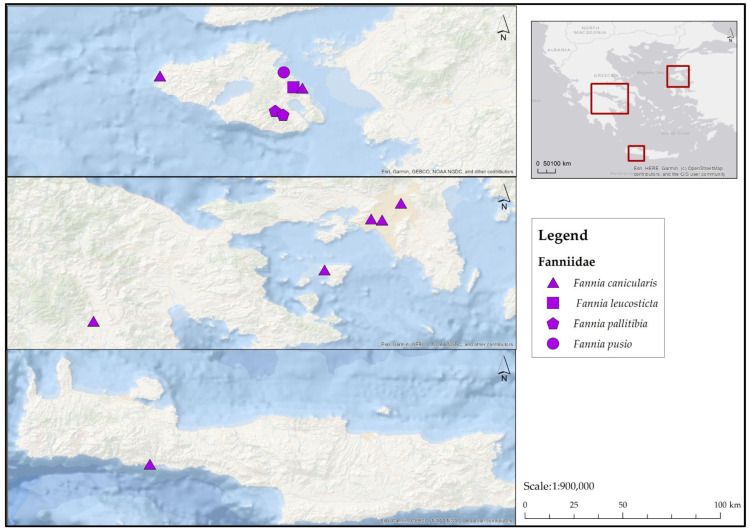
Map depicting collection localities in Greece where Fanniidae were recorded during the present study.

**Family Fanniidae (Figure 4)**
***Euryomma peregrinum* (Meigen, 1826)**
**Distribution in Greece:** Known from mainland Greece without specific localities [43,48].
**Distribution:** Originating in the Neotropical region [48]. In Europe, most common in Mediterranean countries [48].
**Comments:** A cosmopolitan species [94,95,96], also recorded in association with decomposing animal carcasses [97].
***Fannia armata* (Meigen, 1826)**
**Distribution in Greece:** Known from the provinces of Imathia and the Ionian Islands (Corfu) [47].
**Distribution:** Eurosiberian species, reaching up to 1000 m in mountains [48].
**Comments:** Reported as a random visitor of carrion and cadavers [18]. Larvae reared from dung, rotting wood, and fungi; adults secretophagous [48].
***Fannia canicularis* (Linnaeus, 1761)**
**Material examined: GREECE:** AEGINA*: Agion Asomaton, 1♂, 15 May 2013 (M.A.); ARCADIA*: Tripoli, 1♂, 25 March 2023 (S.Z.); ATTIKI: Agia Varvara, 1♀, 11 June 2022 (G.A.); 1♂, 27 February 2023 (G.A.); Agricultural University of Athens, 1♂, 26 May 2021 (G.D.R.); 2♂, 14 June 2021 (G.D.R.); Pefki, 1♂, 11 April 1998 (N.M.N.H.G.); CHANIA: Hora Sfakion, 1♂, 4 June 2023 (S.Z.); LESVOS*: Pirgi Thermis, 1♂, 31 January 2021 (S.Z.); Sigri, 1♀, 29 March 2012 (M.A.).
**Distribution in Greece:** Known from the provinces of Attiki (mainland part) and Crete [3,8,43]. Also reported from “Greek islands”, but without further details [47]. New for Aegina Island (Attiki) and Peloponnese.
**Distribution:** Cosmopolitan and eusynanthropic species [48].
**Comments:** A ubiquitous species of forensic and medical importance [98]. Larvae develop in various types of organic matter, including decomposing carrion and human bodies [50]. The species is also involved in myiasis cases [13]. Currently, this is the only member of the family recorded from Cyprus [35,43,61]. The records from the island of Lesvos verify its presence in the North Aegean Islands as well as Crete.
***Fannia conspecta* Rudzinski, 2003**
**Distribution in Greece:** Known from the provinces of Ioannina and Trikala (Mount Lakmos) [50].
**Distribution:** An uncommon species, scatteredly distributed across Europe [49,50,52,80,81,94,95,96,97,98].
**Comments:** Reported as attracted to carrion during decomposition [50].
***Fannia gotlandica* Ringdahl, 1926**
**Distribution in Greece:** Known from mainland Greece without specific localities [43].
**Distribution:** A rare species, recorded only from a few European countries (Croatia, France, Greece, Sweden, and Great Britain) [43,48].
**Comments:** The species has been reported as saproxylic, emerging from decaying basal heartwood in hollow ash pollards, indicating an association with dead wood microhabitats [99].
***Fannia incisurata* (Zetterstedt, 1838)**
**Distribution in Greece:** Known from mainland Greece without specific localities and Crete [43,47,48].
**Distribution:** Almost cosmopolitan and hemisynanthropic species [48].
**Comments:** A species of medical and hygienic importance [82].
***Fannia latipalpis* (Stein, 1892)**
**Distribution in Greece:** Known from mainland Greece without specific localities [43].
**Distribution:** Widespread in Europe, including the Canary Islands [43,48].
**Comments:** A carrion-associated species. Regarded as occurring mainly in the warmer regions of western and central Europe [100].
***Fannia lepida* (Wiedemann, 1817)**
**Distribution in Greece:** Known from mainland Greece without specific localities [43].
**Distribution:** Widely distributed in the Holarctic region [48].
**Comments:** Recorded as a carrion-associated species on pig carcasses [101].
***Fannia leucosticta* (Meigen, 1838)**
**Material examined: GREECE:** LESVOS*: Pirgi Thermis, 1♀, 21 September 2023 (S.Z.).
**Distribution in Greece:** Known from mainland Greece without specific localities [43,47,48]. New for the North Aegean Islands.
**Distribution:** Almost cosmopolitan and synanthropic [48].
**Comments:** The species has been commonly reared from mink dung and appears to be specialized on dung from domestic carnivorous mammals [102].
***Fannia lustrator* (Harris, 1780)**
**Distribution in Greece:** Known from mainland Greece without specific localities [43].
**Distribution:** Widely distributed in the Palaearctic region, reaching Japan in the East [48].
**Comments:** Eurasian species; more frequent on warmer slopes; numerous records of the species have been reported, often from traps baited with meat [103].
***Fannia monilis* (Haliday, 1838)**
**Distribution in Greece:** Known from the provinces of Magnesia (Mount Pelion) and Crete [43,47,48,51].
**Distribution:** Widespread in the Palaearctic region [48].
**Comments:** Reported from decomposing animal carrion and human cadavers [104].
***Fannia norvegica* Ringdahl, 1934**
**Distribution in Greece:** Known from mainland Greece without specific localities [43].
**Distribution:** Scatteredly distributed across the Western Palaearctic region and recorded from Japan in the Eastern Palaearctic [43,49].
***Fannia pallitibia* (Rondani, 1866)*^▲^***
Material examined: GREECE*: LESVOS*: Radar Agiasou, 1♂, 28 September 2024 (S.Z.); Sanatorio Agiasou, 2♀, 8 October 2023 (S.Z.).
**Distribution:** Recorded from almost all areas of Europe, generally common in meadows and during autumn [48,49]. New for Greece.
**Comments:** Females attracted to decaying meat and excrement [103]. More frequent in lowlands and in autumn [48]. The species has been associated with carrion, but not established as a forensic indicator [105].
***Fannia pusio* (Wiedemann, 1830)*^▲^***
Material examined: GREECE*: LESVOS*: Xampelia Beach, 1♂, 17 September 2022 (S.Z.).
**Distribution:** Originally native to subtropical and tropical regions of the Americas. Currently, introduced to various parts of Africa, Asia, Europe, and Oceania [81]. New for Greece and the Balkans.
**Comments:** A synanthropic species repeatedly recorded from both animal carrion and human cadavers [81].
***Fannia rondanii* (Strobl, 1893)**
**Distribution in Greece:** Known from mainland Greece without specific localities [43].
**Distribution:** Widespread in the Holarctic region [48].
**Comments:** The species has been reared from rotting wood, decaying vegetable matter, owl pellets, and *Delichon* sp. nests [48].
***Fannia scalaris* (Fabricius, 1794)**
**Distribution in Greece:** Known from the province of Magnesia (Mount Pelion) [51].
**Distribution:** Almost cosmopolitan in distribution [48].
**Comments:** A species closely tied to human-modified environments and potentially of sanitary significance, owing to its association with contaminated substrates [106]. Recorded in forensic casework from Central Europe [107].
***Fannia sociella* (Zetterstedt, 1845)**
**Distribution in Greece:** Known from mainland Greece without specific localities [43].
**Distribution:** Widely distributed across Europe, ranging from Ireland and Great Britain through Fennoscandia and northwestern Russia to Spain, Italy, and Greece; also recorded from the Azores; its range extends eastward to China and Japan [103,108].
***Fannia subpubescens* Collin, 1958**
**Distribution in Greece:** Known from mainland Greece without specific localities [43].
**Distribution:** Scatteredly distributed in Europe and also recorded from Alaska in the Nearctic region [48].
***Fannia umbrosa* (Stein, 1895)**
**Distribution in Greece:** Known from mainland Greece without specific localities [43].
**Distribution:** Widely distributed in Europe [48].
**Comments:** Associated with wooded/shrubby habitats; immatures are reported from birds’ nests and sap runs from tree rot holes, with larvae feeding on humic/decaying organic material [109].

**Figure 5:**
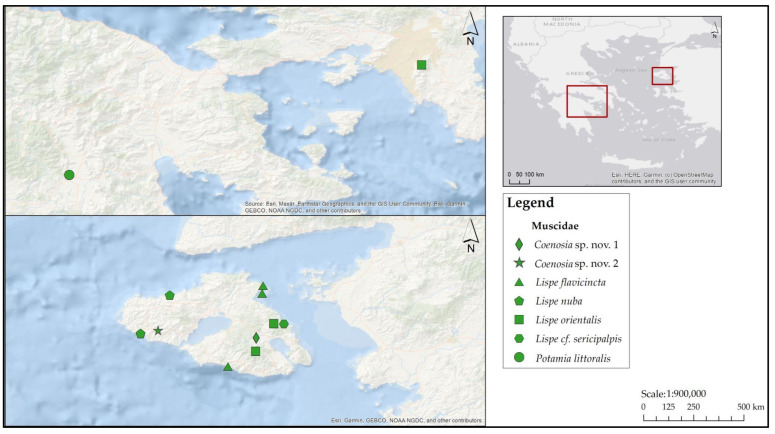
Map depicting collection localities in Greece where newly recorded Muscidae were documented during the present study.

**Family Muscidae (Figure 5)**
***Coenosia* sp. nov. 1*^▲^* (Figure 7D)**
Material examined: GREECE*: LESVOS*: Karini, 1♂, 27 April 2023 (S.Z.).
**Comments:** The classification of this single male specimen terminated near *Coenosia sexpustulata* Rondani, 1866 and *C. sexmaculata* Meigen, 1838 in both of the keys provided by Hennig [47] and Gregor et al. [76]. However, its genitalia are distinctively different. It also differs in various characters (including genitalia) from the more recently described species of the Balkans and nearby areas [58,110,111,112,113] that are not included in those keys. Therefore, it is considered an undescribed species that will be described in a separate paper.
***Coenosia* sp. nov. 2*^▲^***
Material examined: GREECE*: LESVOS*: Pithari Monastery, 1♂, 26 December 2022 (S.Z.).
**Comments:** The classification of this specimen terminated near *Coenosia femoralis* (Robineau–Desvoidy, 1830) in both of the keys provided by Hennig [47] and Gregor et al. [76]. However, its genitalia are different. From the more recently described species of the Balkans and nearby areas that are absent in those keys, it is most similar to *Coenosia persica* Pont and Parchami–Araghi, 2020, another species that terminates near *Coenosia femoralis* when examined against them [113]. However, the two species show differences in leg coloration, leg chaetotaxy, and male genitalia. As such, it is considered an undescribed and probably closely related species which will be described in a separate paper.
***Lispe flavicincta* Loew, 1847*^▲^* (Figure 7G)**
Material examined: GREECE*: LESVOS*: Aspronisi Island, 1♂, 15 September 2022 (S.Z.); Paralia Drotas, 1♂, 21 August 2023 (S.Z.); Palios, 1♀, 30 September 2023 (S.Z.).
**Distribution:** Known from various European countries and reaching Central Asia in the East [112]. New for Greece.
***Lispe nuba* Wiedemann, 1830*^▲^***
Material examined: GREECE*:  LESVOS*: Kalo Limani, 1♀, 20 July 2025 (S.Z.); Skala Eresou, 1♀, 12 August 2023 (S.Z.).
**Distribution:** Widespread in Africa, known only from Egypt and Israel in the Palaearctic region [57]. New for Greece and Europe.
**Comments:** A predatory species, which preys upon emerging adult mosquitoes at the surface of the water in rice field/pond habitats [114].
***Lispe orientalis* Wiedemann, 1824*^▲^***
Material examined: GREECE*: ATTIKI*: Kaisariani Forest, 1♀, 28 May 2025 (G.A.); LESVOS*: Pirgi Thermis, 1♂, 13 July 2020 (S.Z.); 1♂, 19 July 2021 (S.Z.); Sanatorio Agiasou, 1♀, 08 July 2023 (S.Z.).
**Distribution:** Known from various localities in Palaearctic Asia, Russia in Europe, and the Oriental region, being very prevalent in mountainous areas of the latter [57]. New for Greece and the Balkans.
**Comments:** Associated with dirty, organically polluted, typically stagnant waters, especially in habitats enriched with cattle dung, refuse, and occasionally carrion. Frequently encountered near dung-polluted pools (including near cattle sheds and drinking bowls) and have been noted to occur in highly contaminated urban water bodies such as irrigation ditches. Field observations suggest notable rain tolerance. Adults may remain active during prolonged rainfall and hunt dipteran larvae on wet manure substrates [115].
***Lispe* cf. *sericipalpis* (Stein, 1904)*^▲^* (Figure 7C)**
Material examined: GREECE*: LESVOS*: Pirgi Thermis, 1♀, 24 September 2023 (S.Z.).
**Distribution:** In the Palaearctic region, known from a few South European countries, Russia, the Caucasus, the Near East, Central Asia, Pakistan, and China. In the Oriental region, it is very widespread in highland localities [78]. New for Greece.
**Comments:** This particular female matches the characters given for *L. sericipalpis* in the keys of Vikhrev [78,115]. However, there is an absence of posterior setae on the hind tibiae, suggesting that it is probably an aberrant or worn specimen (Vikhrev Nikita *pers. comm.*). Given the absence of a male specimen for genitalia examination or more females, the current identification as *L.* cf. *sericipalpis* is retained.
***Potamia littoralis* Robineau**–**Desvoidy, 1830*^▲^***
Material examined: GREECE*: ARCADIA*: Tripoli, 1♀, 26 March 2023 (S.Z.).
**Distribution:** Hemisynanthropic species, widely distributed in the Holarctic region and marginally entering the Oriental (Myanmar) [76]. New for Greece.
**Comments:** A species closely associated with the nest-building habits of birds [116].

**Figure 6:**
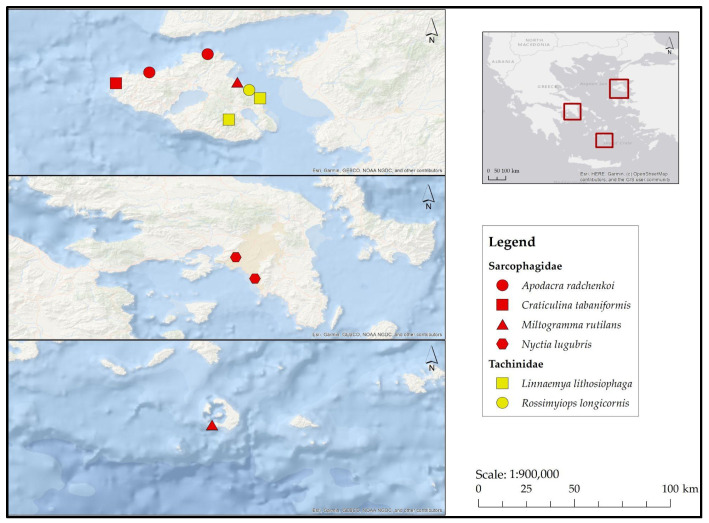
Map depicting collection localities in Greece where newly recorded oestroid families (Sarcophagidae, Tachinidae) were documented during the present study.

**Figure 7:**
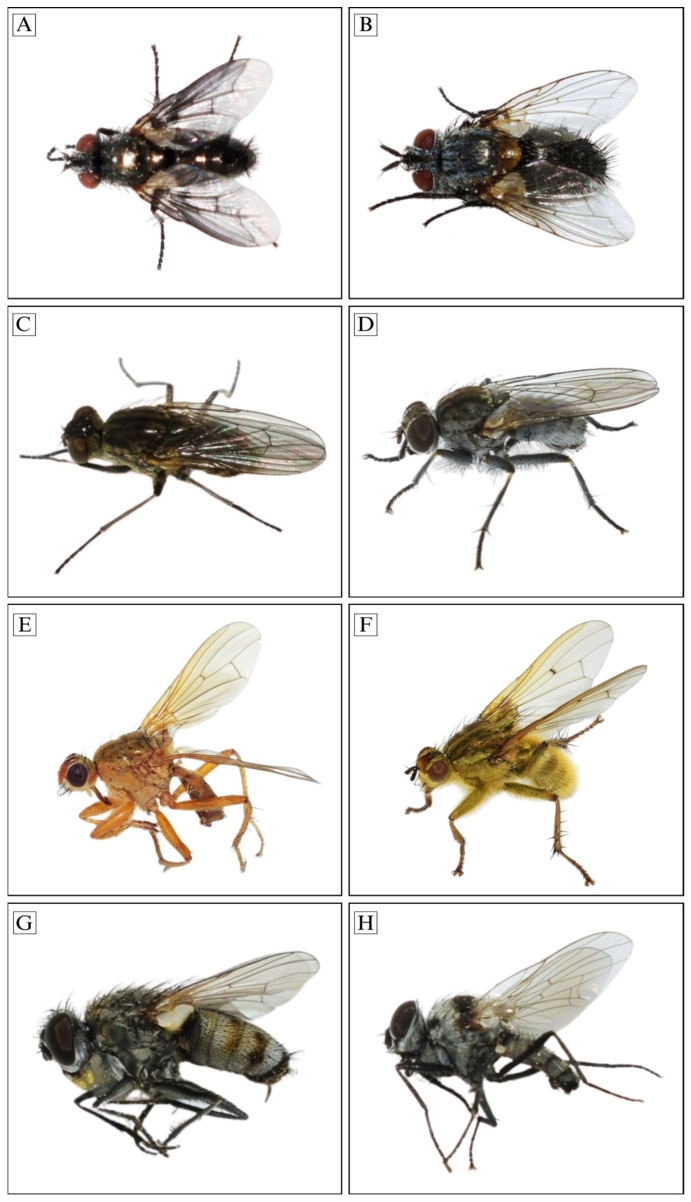
Adult external morphology of selected oestroid and muscoid species: (**A**): *N. lugubris* (Oestroidea: Sarcophagidae) ♂ habitus, dorsal view; (**B**): *Li. lithosiophaga* (Oestroidea: Tachinidae) ♂ habitus, dorsal view; (**C**): *L.* cf. *sericipalpis* (Muscoidea: Muscidae) ♀ habitus, dorsolateral view; (**D**): *Coenosia* sp. nov. 1 (Muscoidea: Muscidae), ♂ habitus, lateral view; (**E**): *S. lutaria* (Muscoidea: Anthomyiidae) ♀ habitus, lateral view; (**F**): *S. stercoraria* (Muscoidea: Anthomyiidae) ♀ habitus, lateral view; (**G**): *L. flavicincta* (Muscoidea: Muscidae) ♀ habitus, lateral view; (**H**): *A. illocata* (Muscoidea: Anthomyiidae) ♂ habitus, lateral view.

**Superfamily Oestroidea**
**Family Sarcophagidae (Figure 6)**
***Apodacra radchenkoi* Verves and Khrokalo, 2015*^▲^***
Material examined: GREECE*: LESVOS*: Archaia Antissa, 1♀, 20 July 2025 (S.Z.); Skala Sikamineas, 1♂, 6 August 2023 (S.Z.).
**Distribution:** Known only from Turkey [54]. New for Greece and Europe.
**Comments:** The biology and ecology of the species remain currently unknown. Records from the island of Lesvos suggest a potential affinity for rocky, near-shore habitats during the summer period.
***Craticulina tabaniformis* (Fabricius, 1805)*^▲^***
Material examined: GREECE*: LESVOS*: Faneromeni Beach, 1♂, 16 August 2023 (S.Z.).
**Distribution:** Widespread in the Palaearctic and Oriental regions [57]. New for Greece.
**Comments:** The genus *Craticulina* has been reported developing in nests of sand wasps, where females larviposit into the stored prey of the wasp. Larvae feed on the provisioned flies and pupate within the burrow in the sand [117].
***Miltogramma rutilans* Meigen, 1824*^▲^***
Material examined: GREECE*: LESVOS*: Xampelia Beach, 1♀, 19 September 2022 (S.Z.); SANTORINI*: Akrotiri–Faros, 1♂, 8 May 2013 (M.A.).
**Distribution:** Widespread in the Palaearctic region, reaching Central Asia to the East [57]. New for Greece.
**Comments:** Considered a psammophilous species [75].
***Nyctia lugubris* (Macquart, 1843)*^▲^* (Figure 7A)**
Material examined: GREECE*: ATTIKI*: Agia Varvara, 4♀, 2 March 2021 (G.A.); 2♂, 8 March 2023 (G.A.); Ellinikon International Airport, 1♂, 6 January 2023 (S.Z.).
**Distribution:** Widespread in the Western Palaearctic region, but restricted to countries around the Mediterranean Sea and the Canary Islands [57]. New for Greece.
**Comments:** Many species of the genus *Nyctia* are considered parasites or specific necrophages of terrestrial snails [118].
**Family Tachinidae (Figure 6)**
***Linnaemya lithosiophaga* (Rondani, 1859)*^▲^* (Figure 7B)**
Material examined: GREECE*: LESVOS*: Vigla Pamfilon, 1♂, 9 September 2022 (S.Z.); Sanatorio Agiasou, 1♂, 12 July 2020 (S.Z.); 1♂, 08 October 2022 (S.Z.).
**Distribution:** Widespread in the Western Palaearctic region [52]. New for Greece.
***Rossimyiops longicornis* (Kugler, 1972)**
**Material examined: GREECE:** LESVOS*: Petalidi Beach, 1♀, 19 September 2022 (S.Z.).
**Distribution in Greece:** Known from the Ionian Islands (Zakynthos), the North Aegean Islands, and Crete [119].
**Distribution:** Bulgaria and Greece in Europe, Israel, Transcaucasia, and Turkey in Asia [120].
**Comments:** The genus *Rossimyiops* is considered an obligate endoparasitoid of Embioptera [119,120]. Until now, *R. longicornis* is known to parasitize three species of Embioptera: *Haploembia megacephala* Kraus, 1911 (doubtful species), *H. solieri* (Rambur, 1842), and an undescribed species of *Haploembia* [120]. The single female was found in an area where only populations of a fourth species, *H. palaui* Stefani 1955, are known. As such, we suggest that *H. palaui* is a possible, new host for the species.

## 4. Discussion

Greece and Cyprus, situated at the intersection of European, Asian, and African biogeographic influences, are recognized as biodiversity hotspots; however, their dipteran fauna remains far from fully explored [9,35,121]. As a result, many families of Diptera that retain significant applied and forensic importance, like Fanniidae and Sarcophagidae [122], lack proper documentation or have only recently been thoroughly reviewed [9]. In the current study, five families of calyptrate flies (Anthomyiidae, Fanniidae, Muscidae, Sarcophagidae, and Tachinidae) belonging to two superfamilies (Muscoidea and Oestroidea) are documented by adding new records to the faunal lists of Greece, Europe, and even the whole of the West Palaearctic region. In addition, the first checklists of the family Fanniidae and subfamily Scathophaginae for Greece and Cyprus are provided.

Anthomyiidae (excluding Scathophaginae) lacks concise documentation in Greece, being only sporadically addressed in a few papers and databases with a total of 93 species recorded [43,44]. *Anthomyia illocata* is a synanthropic, characteristically patterned species that is widespread in the East Palaearctic, Oriental, and Australasian regions. Nevertheless, it has not been officially reported outside these regions, even though numerous American and European records of this species occur in citizen science projects and databases like GBIF [123] and iNaturalist. As such, its inclusion here represents the first official record for the whole of the West Palaearctic region, raising the total number of recorded species to 94.

Within Anthomyiidae, the subfamily Scathophaginae is another group that has been inadequately and scarcely documented in Greece, with only three species recorded [3,7,8,43,45,46]. This study provides the first checklist for the family, and, like Fanniidae, a revision of the available literature and examination of fresh and museum material allowed the compilation of the first Greek checklist for the family. Four species were found to occur in Greece, from which *S. lutaria* is newly added, while the validity of *S. ochrocephala* is ambiguous and must be tested via examination of its type specimens. In addition to the Greek checklist, a similar one was prepared for the island of Cyprus. Based on the available literature, four species of *Scathophaga* are considered to occur in Cyprus [35]. However, only one of those records (*S. stercoraria*) can be accepted as valid, as museum material was examined and verified, while two species (*S.* ?*furcata*, *S. taeniata*) remain provisionally reported, with the identification of one of them (*S.* ?*furcata*) being ambiguous.

Fanniidae, like Anthomyiidae, have been occasionally documented in Greece, with 17 species being recorded [3,8,43,47,48,49,50,51]. In this study, existing literature data, museum specimens, and newly collected material were examined, and the first checklist for this group in the Greece/Cyprus region is provided. As a result, 19 species were found to occur, one of which (*F. pallitibia*) constitutes a new record for Greece, and one (*F. pusio*) for the Balkans. Published data for Cyprus were also examined, with only one species (*F. canicularis*) recorded.

Muscidae is well documented in Greece, with 160 species recorded in a relatively recent checklist [39] and a few additional papers [42,58,59,60]. However, examination of newly collected material by the authors led to the addition of another seven species to the Greek muscid list, raising the total number to 167. Two of these constitute new, undescribed taxa of the genus *Coenosia*, four (*L. flavicincta*, *L. orientalis*, *L.* cf. *sericipalpis,* and *P. littoralis*) are new for the country, and one (*L. nuba*) is new for Europe.

The family Sarcophagidae is only partially documented in Greece, with recent coverage provided by a checklist of Greek species of the genus *Sarcophaga* [9], while records of other sarcophagid genera in Greece are otherwise dispersed among bibliographic resources and databases, together providing a total number of 109 species [43,54,55,56,57]. This study lists four species belonging to the subfamilies Miltogramminae and Paramacronychiinae, raising the total number of recorded sarcophagid species to 113. Three of these species (*C. tabaniformis*, *M. rutilans,* and *N. lugubris*) are recorded from Greece for the first time, while the fourth (*Ap. radchenkoi*) is new for Europe.

Tachinidae is well documented in Greece, with 347 species being reported through various references and databases [38,43,52,53], even though no official checklist seems to exist for the country. The current study adds another species (*Li. lithosiophaga*) to this list, raising the total number to 348, and documents ecological comments for *R. longicornis*.

Overall, the Greek and Cypriot oestroid and muscoid fauna is richer than previously thought, but substantial gaps still remain in both taxonomic and geographic coverage. Continued faunistic surveys, particularly in under-sampled habitats and regions, combined with integrative approaches and inclusion of molecular data, will therefore be essential to resolve taxonomic uncertainties, describe new taxa, and refine distributional ranges. Such efforts will improve the resolution of biogeographic and ecological analyses and strengthen the role of calyptrate flies in applied entomological research in Greece and Cyprus.

## 5. Conclusions

The present contribution expands the known composition of the Greek and Cypriot calyptrate fauna by validating the presence of the subfamily Scathophaginae in Cyprus and documenting various species from five muscoid and oestroid families, including 16 species reported for the first time from Greece. Future work should prioritize the evaluation of phenology, habitat associations, and functional traits within these groups. Finally, many of the taxa reported here belong to taxonomic groups with distributions extending beyond Greece and Cyprus; consequently, these records also create opportunities for faunistic and distributional comparisons across the Mediterranean and adjacent regions.

## Figures and Tables

**Table 1:** List of collection localities for the specimens examined in the present study.

Locality N°.	Country	Region (County/Island)	Locality	Coordinates	Ecotype
1	Cyprus	Lemesos	Peak of Mountain Troodos	34°55′54.1″ N, 32°51′59.0″ E	Mountainous
2		Nicosia	Linou	35°04′31.8″ N, 32°54′59.0″ E	No data *
3	Greece	Aegina	Agion Asomaton	37°45′01.8″ N, 23°26′26.9″ E	Urban
4			Kavouropetra	37°46′04.4″ N, 23°27′10.1″ E	Urban
5		Anafi	Helicodrome	36°21′25.2″ N, 25°46′19.2″ E	Phrygana
6		Andros	Zaganiaris	37°46′44.0″ N, 24°52′45.5″ E	Phrygana
7		Arcadia	Tripoli	37°30′51.4″ N, 22°22′30.9″ E	Urban
8		Attiki	Agia Varvara	37°59′12.3″ N, 23°39′18.1″ E	Urban
9			Agricultural University of Athens	37°58′55.2″ N, 23°42′21.6″ E	Urban
10			Alsos Syggrou	38°03′53.4″ N, 23°48′54.7″ E	Pine Forest
11			Diomedes Botanical Garden	38°00′25.2″ N, 23°38′34.8′′E	Phrygana
12			Ellinikon International Airport	37°53′09.6″ N, 23°44′42.0″ E	Urban
13			Kaisariani Forest	37°57′32.1″ N, 23°47′56.3″ E	Pine Forest
14			Pefki	38°03′33.2″ N, 23°47′35.0″ E	Urban
15		Chania	Hora Sfakion	35°12′07.2″ N, 24°08′13.2′′ E	Urban
16			Kefali	35°23′18.9″ N, 23°54′25.5″ E	Olive groves
17			Omalos I	35°22′19.1″ N, 23°53′39.2″ E	Mountainous
18			Omalos II	35°18′57.6″ N, 23°54′53.2″ E	*Zelkova abelicea* forest
19		Chios	Gridia	38°12′55.8″ N, 26°06′05.8”E	No data*
20			Pityos	38°29′22.9″ N, 26°03′42.5″ E	Phrygana
21			Vessa	38°16′34.3″ N, 26°00′58.7″ E	Phrygana
22		Evros	Dadia	41°01′46.6″ N, 26°09′04.7″ E	No data *
23		Kea	Mylopotamos	37°38′34.8″ N, 24°19′28.6″ E	Phrygana
24		Korinthia	Lechaio	37°56′06.6″ N, 22°51′35.6″ E	Urban
25		Lakonia	Agios Stefanos	36°38′14.7″ N, 23°01′11.8″ E	No data *
26		Lesvos	Archaia Antissa	39°17′22.6″ N, 26°01′02.7″ E	Phrygana, Grasslands
27			Aspronisi Island	39°17′53.3″ N, 26°26′00.1″ E	Phrygana
28			Chestnut Forest I	39°03′18.0″ N, 26°23′51.7″ E	Chestnut Forest
29			Chestnut Forest II	39°03′17.0″ N, 26°23′50.0″ E	Chestnut Forest
30			Chestnut Forest III	39°03′45.0″ N, 26°23′30.0″ E	Chestnut Forest
31			Drota beach	39°00′14.0″ N, 26°16′42.1″ E	Sandy beach
32			Faneromeni Beach	39°14′18.8″ N, 25°51′38.2″ E	Sandy beach
33			Kalloni Saltpans	39°12′33.0″ N, 26°14′37.0″ E	Wetlands
34			Kalo Limani	39°17′28.4″ N, 26°02′34.3″ E	Wetlands
35			Kamaria Pamfilon	39°09′36.5″ N, 26°30′33.5″ E	Olive groves
36			Karini	39°07′10.6″ N, 26°23′30.4″ E	Riverbank, Olive groves
37			Lisvori	39°06′09.0″ N, 26°11′59.0″ E	Phrygana
38			Moria	39°07′40.0″ N, 26°30′53.0″ E	Olive groves
39			Mytilene	39°05′57.5″ N, 26°33′14.9″ E	Urban
40			Palios	39°19′46.3″ N, 26°25′13.2″ E	Sandy beach
41			Panagia Mirsiniotissa	39°15′10.5″ N, 26°11′37.4″ E	Pine forest
42			Petalidi beach	39°12′20.7″ N, 26°29′07.1″ E	Rocky beach
43			Petrified Forest Park “Bali Alonia′′	39°12′24.8″ N, 25°54′08.3″ E	Phrygana
44			Pirgi Thermis	39°10′32.0″ N, 26°30′15.8″ E	Urban, Olive groves, Grasslands
45			Pithari Monastery	39°09′49.5″ N, 25°57′40.2″ E	Oak forest
46			Radar Agiasou	39°03′02.5″ N, 26°23′23.9″ E	Oak and Chestnut mixed forest
47			Sanatorio Agiasou	39°03′57.6″ N, 26°23′24.0″ E	Chestnut forest
48			Sigri	39°13′38.6″ N, 25°51′30.2″ E	Olive groves
49			Skala Eresou	39°08′12.3″ N, 25°55′30.0″ E	Sandy beach
50			Skala Sikamineas	39°22′33.4″ N, 26°17′27.7″ E	Rocky beach, Olive groves
51			Vigla Pamfilon	39°10′02.2″ N, 26°32′13.2″ E	Sandy beach
52			Xampelia Beach	39°14′36.5″ N, 26°25′46.6″ E	Rocky beach
53		Limnos	Moudros	39°50′22.6″ N, 25°18′36.7″ E	Phrygana
54			Panagia	39°59′25.1″ N, 25°23′30.1″ E	Phrygana
55			Plaka–Panagia	39°59′24.0″ N, 25°24′48.6″ E	Phrygana
56		Pieria	Oropedio Olymbou	40°05′40.9″ N, 22°22′08.4″ E	Alpine
57			Robola Olymbou	40°06′33.8″ N, 22°23′37.0″ E	Alpine
58		Santorini	Akrotiri–Faros	36°21′24.8″ N, 25°21′41.8″ E	Phrygana

* “No data” denotes that ecotype/habitat information for the respective locality was unavailable (i.e., not recorded at the time of collection, not ascertainable, or absent from the original source).

## Data Availability

The specimens listed in this study are deposited in the private entomological collections of the contributing authors (G.D.R., S.Z., G.A.), the National Museum of Natural History Goulandris (N.M.N.H.G.), and the Melissotheque of the Aegean (M.A.) (University of the Aegean) and are available upon request. The datasets generated during this study are available from the corresponding author upon reasonable request.
